# Handling students’ misbehaviors in crowded classrooms: the nursing faculty members’ experiences

**DOI:** 10.1186/s12909-023-04692-5

**Published:** 2023-09-28

**Authors:** Shahin Salarvand, Reyhaneh Niknejad, Razak M. Gyasi

**Affiliations:** 1 Hepatitis Research Center, Faculty of Nursing and Midwifery, Lorestan University of Medical Sciences, Khorramabad, 6814993165 Iran; 2 Community health research center, Isfahan(Khorasgan)Branch, Islamic Azad University, Isfahan, Iran; 3 African Population and Health Research Center, Nairobi, Kenya; 4 Faculty of Health, National Centre for Naturopathic Medicine, Southern Cross University, Lismore, NSW Australia

**Keywords:** Teachers, Students’ misbehaviors, Classroom management, Nursing

## Abstract

**Introduction:**

The ability of teachers to organize classes and manage the behavior of their students is critical in achieving positive educational outcomes. The aim of this study was to explain the experiences of nursing faculty members in managing disruptive behaviors in the classroom.

**Methods:**

The study adopted descriptive explanatory qualitative study design and provided an avenue to explain the experiences of nursing faculty members in managing disruptive behavior in the classroom Participants were included via the purposive sampling. In-depth and semi-structured interviews were used to collect data. The content analysis presented by Graneheim and Lundman was used to analyze the data. The present study utilized four strength criteria, including credibility, confirmability, transferability, and dependability.

**Results:**

The finding were presented using five themes that emerged from 350 open codes, including managing disruptive behavior in the classroom, guiding the disruptive student, trying to increase learning, and making the class more interesting, setting the rules and regulations of the class with sub-categories.

**Conclusions:**

Participants cited strategies that they enabled to understand the cause of misbehavior and implement strategies to modify students’ misbehaviors by creating a safe and healthy climate to nurture effective learning by students.

**Supplementary Information:**

The online version contains supplementary material available at 10.1186/s12909-023-04692-5.

## Introduction

The main goal of education is to train students to be good citizens, transform the society in which they live, and contribute to building a cleaner, more sustainable community [1]. A desirable academic environment should provide learning as a core subject and empower students to discover the appropriate value system that can guide them in self-awareness and developing a sense of national and global consciousness [2]. The classroom is a social organization for education, and the presence of order is very instructive to enhancing the effectiveness and efficiency of the teacher’s teaching. A teacher who can effectively manage the classroom creates a suitable learning environment for students [3]. To achieve teaching and learning goals, classroom management becomes essential [4]. If a teacher cannot manage his/her classroom using multiple appropriate techniques, his/her teaching process may not be effective [3].

Given that behavior is one of the social aspects of the classroom environment of significant importance [5], the frequency and prevalence of educational misbehavior is a major problem in university classrooms, and its magnitude has increased dramatically over the past two decades [6]. Due to the high prevalence of educational misbehavior among students in crowded classrooms, identifying the types and the prevalence of educational misbehavior should be considered by educational authorities and faculty members in the universities [6]. In this study, disruptive classroom behavior means indiscipline, misbehavior, and lack of attention that can disrupt the learning process. These student’s misbehaviors can have far-reaching detrimental effects on the experience and emotional state of teachers and students, hinder the achievement of teaching goals, and reduce the overall effectiveness of learning for all of class [7, 8]. Classroom management is a term that scholars use to describe how to ensure that the teaching process in the classroom runs smoothly, even when students behave in a disruptive manner. This term refers to preventing disruptive behaviors, which is likely one of the most challenging aspects of teaching for many teachers [3]. For some teachers, it is problematic and intolerable to maintain an orderly and productive learning environment in the classroom [4]. These challenges may cause teachers to leave the profession [9]. The costs of disruptive classroom behaviors can be accounted for in terms of negative impacts on student learning, school climate, and teacher well-being [10, 11]. As such, preventing, controlling, and reducing disruptive behaviors are critical skills for anyone hoping to teach effectively in face-to-face and one-to-many teaching situations. Competence in establishing and maintaining order, involving students, and gaining their trust, respect, and cooperation are essential aspects of classroom management [7]. This, in turn, is a necessary topic in educational research [12].

Classroom management can be defined as a set of techniques and activities for creating and maintaining an effective learning environment that provides a quality training climate, improves class working conditions, and eliminates any distractions that may arise [13]. Teachers who engage in effective classroom management practices utilize a range of strategies to increase appropriate behavior and decrease inappropriate behavior which varies based on the complexity and severity of the behavior [14, 15]. These strategies include maximizing structure, setting and reinforcing expectations, involving students, and recognizing appropriate and responding to inappropriate behavior [16]. Lacking these skills, teachers often struggle to maintain order and spend more time managing misbehavior than teaching [17]. Therefore, the development of classroom management skills is becoming increasingly critical [18].

Unfortunately, although positive classroom management skills are necessary to maximize students’ academic and social achievement, many teachers have indicated that behavior management is a skill they are least prepared for doing it [19, 20]. A review of the literature shows that having skills in classroom management is a concern for teachers that should be considered [21–24]. As mentioned above, worse still, when teachers are not well-prepared to manage a classroom, they are likely to be dissatisfied with their job and leave the profession [25]. Of course, teachers usually have their own classroom management strategies for dealing with student misbehavior [26]. Furthermore, classroom management approaches may be contextual or culturally different, but these approaches are more likely to be dependent on local circumstances than on cultures [27]. In order to reduce this misbehavior, suitable disciplinary preventive measures based on culture and important measures for teaching and controlling the students’ behavior in the classroom are necessary [6]. In this study, the management of disruptive behavior has been studied in a face-to-face/in-person classroom and, a crowded class means a class with 30 to 50 students [26]. We conducted this study in nursing faculties. Due to the nursing shortage and the high recruitment demand in the nursing profession in Iran, the classes in nursing schools are among the most crowded classrooms. In addition, the first and second authors are nursing educators. They are interested in and familiar with the study context. Several reviews and research studies have focused on classroom management in Iran [3, 28–32] However, these studies have not used qualitative approaches to provide rich insights on a specific subject. To our knowledge, this is the first original study in this field to unearth the experiences of faculty members in managing disruptive behavior in the classroom. This study employed a qualitative approach and content analysis to understand the experiences of teachers. One of the consequences of qualitative research is the gathering of information in areas where knowledge and/or a deep understanding of people’s experiences are insufficient [33]. Therefore, our study adopted a naturalistic paradigm. Furthermore, the identification of these behaviors significantly influences the learning-teaching process and provides an important picture of the way in which such behaviors are dealt with and reflected upon by the teachers [6]. The content analysis will show how researchers conceptualized the possible responses of teachers, which can play an important role in classroom management and student discipline. This study aimed to explain the experiences of nursing faculty members in managing disruptive behaviors in the classroom.

## Methods

### Study design and sampling techniques

This is explanatory qualitative research using a content analysis approach for data analysis. This study was conducted at the Lorestan University of Medical Sciences as a western province of Iran, which includes four nursing faculties in four different cities. The main faculty is placed in Khorramabad. In sum, these nursing faculties have 46 nursing faculty members. Participants were recruited by purposive sampling according eligibility criteria. The participants were nursing faculty members/teachers and had work experience of at least five years. The sampling was ended after achieving data saturation.

### Data collection

Data were collected from 1 July to 31 October 2022 using face-to-face semi-structured in-depth interviews. Data saturation was achieved by 11 participants with 15 interviews. The interviewer(SS) established a relationship prior to study commencement. She is working in the same nursing faculty. She explained her personal goals and reasons for doing the research to the participants. The interviews were conducted in a calm and quiet environment such as an office room/workspace/free space of the faculty that were convenient to the participants. The informed consent form was completed by all participants before the interviews. Interviews averagely lasted 30 to 90 min. Upon the agreement of the interviewees, the interviews were audio-recorded. The extraction of primary codes and interviews were conducted simultaneously. Thus, the next interview was conducted after analyzing the previous interviews and extracting the primary codes. During the interview, several open questions in the collection of information were applied. The main question was “What are your experiences in counter with the students’ misbehaviors in your classroom management? How do you manage students’ misbehaviors/disruption in your classroom?”. Respondents were questioned for further details, questions were asked to obtain rich and in-depth information (Table 1).

**Table 1:** The topic guide of study/Questions asked in the interviews

Questions
What are your perception/experience of students’ misbehaviors in your classroom?
What are your memories of students’ misbehaviors in your classroom?
How do you manage students’ misbehaviors/disruption in your classroom?
What’s your idea about the management of disruptive behaviors/misbehaviors in the classroom?
Describe your experience of starting and continuing a classroom teaching session.
please explain more….

Face-to-face interviews with participants were conducted by the first author. It is worth noting that interviews were conducted in Persian/Farsi language given that the study was carried out in the Iranian context. The transcripts were later translated to English. The second author (RN) had an interest in the research subject and transcribed the interviews verbatim. Transcription was performed alongside data collection. That is, the participant’s recorded statements were played repeatedly and transcribed verbatim after each interview. The analysis process was conducted by The first author (S.S.).

The first author(SS) is an experienced researcher and a nursing faculty member and she worked on several qualitative content analysis as the main author and has a Ph.D. degree in nursing education. The second author(RN) is an administrator in the community health nursing research center and holds an MSc degree in operating room Nursing. The third author (RMG) is a Medical Gerontologist and senior scientist whose research focuses sub-Saharan Africa and other low- and middle-income countries.

### Data strength

The four criteria proposed by Lincoln and Guba [33], including dependability, transferability, confirmability, and credibility, were applied to ensure the rigor of the study. To obtain credibility; the extracted codes were sent to the study participants. Their confirmation indicated the validity of the codes and ensured that the data analyst understood the participants’ experiences correctly (member check). In addition, peer check was applied, where the results and extracted codes were shared with other qualitative research experts for extensive review and validation (MS.M) (peer check) and the findings were revised based on the expert review and the comments provided. The interviews and primary data analysis duration were prolonged for five months. This prolonged engagement was crucial to allow the researchers to be immersed deeply in the site/study context in order to build trust with the participants and gain a deep understanding of the culture [34]. In addition, the researcher(SS), who conducted interviews and primary data analysis, put aside her preconceived notions, experiences, and thoughts about the subject (bracketing) and avoided reviewing the literature before collecting data to avoid researcher’s bias during the interviews and data analysis. In the present study, crucially, triangulation occurred at the level of the researcher and, Investigator triangulation, so that each team member presented different comments and we achieved an integrated perspective. To ensure confirmability, researchers applied bracketing and documented the stages of the study in detail to enable an audit trail. To ensure dependability, two researchers were involved in the data analysis process (SS and RMG). In addition, the coding process was revised by the second author (RN). We asked the two external experienced qualitative research experts to revise and categorize the coding (MS.M and NB). Given this study was conducted in a certain discipline and geographic area with a specific culture. Since in qualitative studies, there is no definite “truth”. In general, we were keenly interested an in-depth understanding of a specific issue and in showing different perspectives rather than a singular truth and generalization [35]. Although we tried to increase the generalizability of the findings by implementing rigor components, in qualitative research, we depend on the context and that we sought context-based results. Therefore, transferability was made possible by selecting participants with different demographic characteristics. Moreover, we provided a thick description to describe not only the behavior and experiences but also their context so that the behavior and experiences are meaningful to the reader. In other words, we helped other researchers in different locations achieve similar/same results by addressing rigor, such as audit trails and other items. We applied multiple coders to interpret data. The external reviewer, and conducted peer and member checking, in order to prevent bias in this qualitative inquiry.

We addressed reflexivity as a multidimensional and ongoing practice throughout our research process. Reflexivity can be defined as a set of collaborative, multifaceted, and continuous practices through which qualitative researchers self-consciously appraise, evaluate, and critique how their subjectivity and context impact the research processes [36]. We also emphasized the multifaceted nature of reflexivity; it contains critical addressing of personal, interpersonal, methodological, and contextual agents that impact the study being done and we explained them as follows; In personal reflexivity, the researchers were conscious of their values, life experiences, and beliefs to identify their influence on how they collected and interpreted their research data. In other words, a reflexive process is a suitable tool that can help researchers validate qualitative inquiry findings e.g., As mentioned above, the authors have been teaching in the classroom for many years and have experienced the concern of classroom management and guiding the classroom toward educational aims. Therefore, the first author, who conducted the interviews, put aside his preconceptions to understand the participants’ experience(bracketing), She avoided asking questions that only led respondents in one particular direction, and the research team considered rigor items over the data analysis process.

Interpersonal reflexivity refers to how the participants interpret interview questions, how the participants’ unique perspectives, and how the relationships among the researchers impact the research process [36]. In the present study, there was a transactional relationship between the research team and participants that resulted in 15 interviews with 11 participants to clarify and understand participants’ perspectives. We achieved the participants’ confirmation about extracted data by member checking. In addition, in the present study, the first author as the interviewer is working in the main faculty. She does not have any organizational relationships with other faculties. There was a collaborative interrelation between the research team and we took the assistance of external researchers to revise our work.

Methodological reflexivity refers to the researchers asking themselves how to make methodological decisions and their implications [36]. We focused on the meaning of these decisions and ensuring that they were ethical, accurate, and aligned.

Contextual reflexivity refers to the researchers asking themselves how the aspects of context influence the research and involved people [36]. We described the research context and participants’ characteristics which may have influenced on the study findings.

### Data analysis

The content analysis approach designed by Graneheim and Lundman was used [37]. At the end of each interview, the recorded voice was transcribed verbatim, then each transcript was studied several times to understand the participants’ experiences and impressions to identify meaningful units. All-important and relevant phrases or information were underlined to highlight the statements of interest. For instance, a participant said: “A student exhibited an anti-social behavior by stepping on his classmate’s chair and then pushed it forward…I looked at him silently but he realized that his behavior was awkward …”. The significant unit that was underlined part is the most important phrase extracted from the respondent’s statement. Then the meaningful units were summarized and condensed, and the initial codes emerged. In this instance, warning to the student with non-verbal gestures is an initial code. This initial code with other similar codes were categorized and formed a subcategory of “Purposeful non-verbal gestures in shutting down the disruptive behavior”. S.S. carefully considered the original codes and categorized them into subcategories according to their similarities in concepts. Using this inductive process, similar sub-categories were assigned to the categories. For instance, due to similarity in concept, three subcategories such as; “Purposeful non-verbal gestures in shutting down the disruptive behavior”, “Avoiding making quick judgments” and “Experiencing the effectiveness of advice with humor” were categorized under a higher level of abstraction entitled: “The reaction to the disruptive behavior”. Then this category with similar categories formed a higher level of abstraction category named theme entitled “Managing disruptive behaviors/misbehaviors in the classroom”. This coding process and the emergence of the themes were reviewed and discussed by the second author (RN) and by an experienced researcher(MS.M) with SS. Finally, categories were determined as the expression of the implicit content of the transcripts. In the present study, purposive sampling was applied to include the participants of the target population. It is worth noting that we used Persian/Farsi language in the interviews because this study was conducted in the Iranian context. As in similar studies, after ending the research, the first author wrote the manuscript in English.

### Ethical consideration

The study protocol was reviewed and approval by the Research Ethics Committee of the Lorestan University of Medical Sciences, (Approval Code No. IR.LUMS.REC.1394.2137). Following this, the consent of the persons in charge of the hospitals concerned and the informed written consent of the participants was obtained. In addition, participants were informed of the study objectives and methodologies of the study, including the need to record interviews, and their rights, including confidentiality, anonymity, the assignation of a private code to each participant, and the unfettered right to opt out of the study.

## Results

**Fig. 1:**
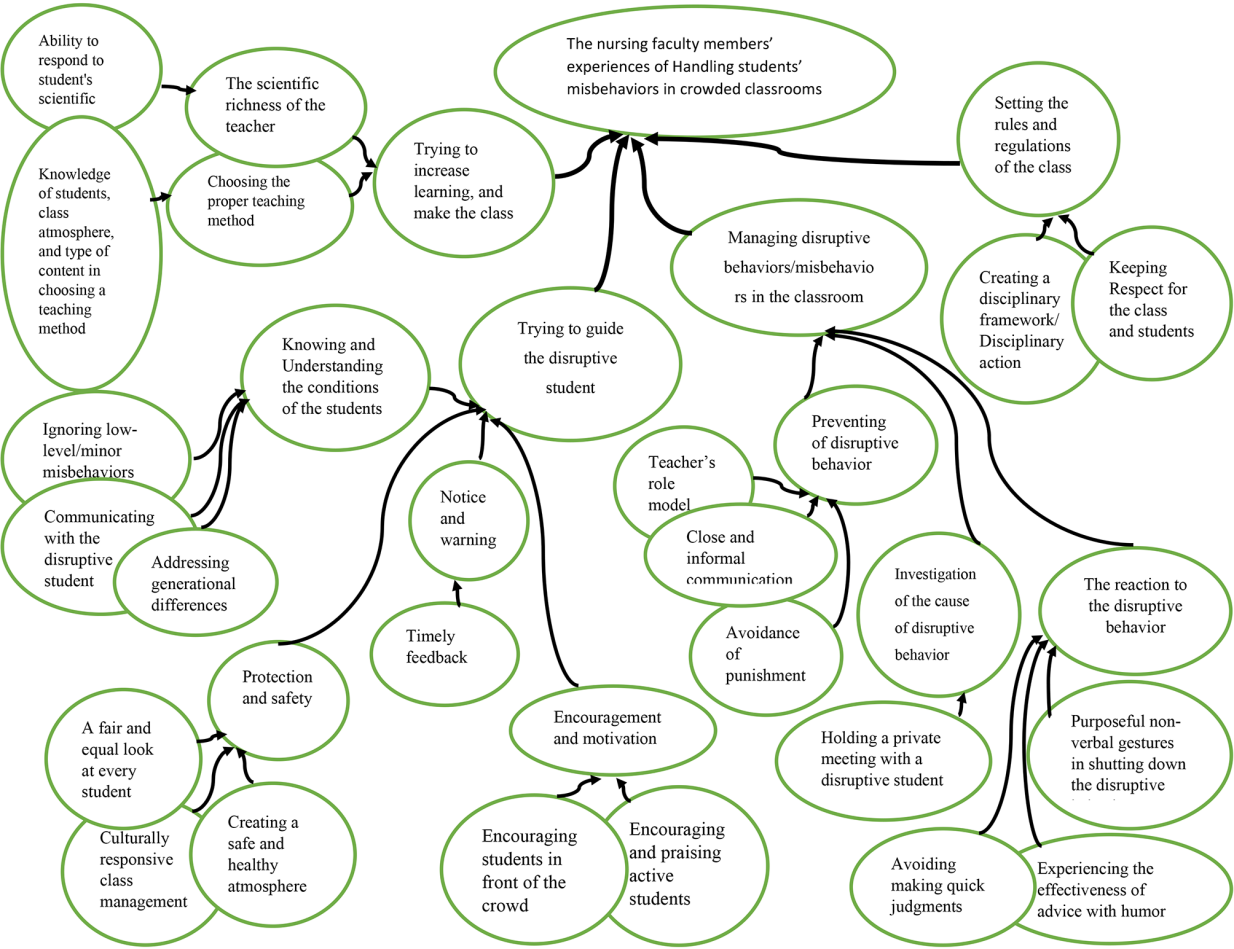
Coding tree of the study

This study explored the experiences of nursing faculty members in managing disruptive behaviors/misbehaviors in the classroom. The participants comprised of six females and five males with mean age is 42.5 years old. The results of the present study included four themes emerged from 350 codes. These include managing disruptive behaviors/misbehaviors in the classroom, trying to guide the disruptive student, trying to increase learning, and make the class more interesting, and setting the rules and regulations of the class with sub-categories (Table 2; Fig. 1).

**Table 2:** Subcategories, Categories and Themes of data analysis

Sub-categories	Categories	Themes
Purposeful non-verbal gestures in shutting down the disruptive behavior	The reaction to the disruptive behavior	Managing disruptive behaviors/misbehaviors in the classroom
Avoiding making quick judgments		
Experiencing the effectiveness of advice with humor		
Holding a private meeting with a disruptive student	Investigation of the cause of disruptive behavior	
Teacher’s role model	Preventing of disruptive behavior	
Close and informal communication		
Avoidance of punishment		
Addressing generational differences	Knowing and Understanding the conditions of the students	Trying to guide the disruptive student
Ignoring low-level/minor misbehaviors		
Communicating with the disruptive student		
A fair and equal look at every student	Protection and safety	
Culturally responsive class management		
Creating a safe and healthy atmosphere		
Timely feedback	Notice and warning	
Encouraging students in front of the crowd	Encouragement and motivation	
Encouraging and praising active students		
Ability to respond to student’s scientific questions	The scientific richness of the teacher	Trying to increase learning, and make the classroom more interesting
Knowledge of students, class atmosphere, and type of content in choosing a teaching method	Choosing the proper teaching method	
Keeping Respect for the class and students		Setting the rules and regulations of the classroom
Creating a disciplinary framework/ Disciplinary action		

### Managing disruptive behaviors/misbehaviors in the classroom

Faculty members tried to control and manage disruptive behavior in the classroom with several approaches. One of these approaches was the reaction to the disruptive behavior, where most of the participants referred to targeted non-verbal gestures in shutting down the disruptive behavior.

“…*if a student comes late, it’s not like I don’t let him come… the moment a student comes late, I try to be silent so that the student can sit down… and this causes the others to warn him that, … sit down quickly!!… But it is not usual to tell the student to go back*” P.10.

Non-verbal feedback such as silence for student delay and temporary interruption of teaching:

“…*By being silent and interrupting teaching, I understand the person and the class that being late is not a good manner…*” P.1.

Participants mentioned interrupting teaching and sitting down to control the class as an effective strategy.

*“…instead of directly dealing with the disruptive behavior in the class, I stopped teaching for two minutes and sat down, and this method has worked so far… and the disruptive behavior has not been stopped or repeated”* P.4.

Avoiding making quick judgments.

*“…I try not to judge too soon about the students’ disruptive behavior or their delay….and at first, I avoid direct warning to the student in the class and give a general warning until I have to check the cause.“. P. 9*.

*“If the re-establishing disruptive behavior being occurred, I will give a direct warning to the student”. P. 2*.

Experiencing the effectiveness of advice with humor.

*“Coercive confrontations usually do not work if the student’s personality is crushed in front of the crowd in the classroom… I talk to them with humor and advice”. P. 1*.

Investigation of the cause of disruptive behavior.

Faculty members stated that they considered it necessary to investigate the cause of disruptive behavior. Holding a private meeting with a disruptive student and investigating the cause of the student’s tardiness, lack of motivation, fatigue, and problems in the student’s life were some issues they mentioned.

*“We see the well-groomed appearance of the student and are unaware of the problems in her/his life … I set up a private meeting with the disruptive student and would like to hear what she/he has to say before taking any action.” P. 9*.

Preventing of disruptive behavior: Some participants stated teacher role modeling and close and informal communication are the silent languages to warn the disruptive student.

*“I am willing to communicate a simple and informal relationship with the student, and most importantly, the student should learn from my behavior and gesture. For example, if I am late, I can no longer remind the student to follow the discipline” P.11*.

### Trying to guide the disruptive student

Subcategory of Knowing and Understanding the conditions of the students, protection and safety, notice and warning, and encouragement and motivation. Faculty members stated that in order to manage disruptive behavior, they tried to communicate with the approaches of addressing generational differences, ignoring minor disruptive behaviors, and communicating with the disruptive student.

Ignoring low-level/minor misbehaviors due to the belief that it is impossible for students couldn’t talk in the classroom, and ignoring these disruptive behaviors, were expressed by the professors. *“A student cannot be silent for the whole class” P.1*.

Participants stated that communicating with the disruptive student and addressing generational differences.

*“…I pay attention to generational differences, this new generation has different demands and expectations*…” P.10.

Participants said to try to hold Protected and a safe class atmosphere, including Culturally responsive teaching, creating a safe and healthy atmosphere, Notice and warning, encouragement, and motivation. Moreover, they emphasized a fair and equal look at every student.

*“…I try to have a fair and equal look at every student…” P.11*.

*“…I pay attention to ethnic and cultural sensitivities in classroom management” P.7*.

*“My effort is to provide a safe and healthy atmosphere for better learning” P. 3*.

“*I give feedback to the disruptive student on time/timely and make him aware that I am paying attention” P.5*.

This subcategory consisted of encouragement and motivation, which included encouraging students in front of the crowd and encouraging and praising active students.

“…*I encourage the student in front of the crowd in the classroom … and I say that she will get a positive … and I record it in my sheet*…”. P.1.

### **Trying to increase learning, and make the classroom more interesting**

This theme included the subcategories of the academic richness of the teacher, and choosing the appropriate teaching method.

*“I ask the disruptive student about the material presented in the class … in this way, she/he gets some attention*”. P.8.

Most participants said the teacher’s scientific richness and ability to respond to students’ scientific questions are essential to successful classroom management.

“…*I like my students consider me an up-to-date teacher who can be accountable so that my class sessions become attractive* …” P. 6.

Participants stated choosing an appropriate and effective teaching method based on knowledge of students, class atmosphere, and the type of content in choosing a teaching method. They said these items make the classroom attractive.

*“…According to the students’ preferences and the class atmosphere, the type of content, and the level of the students, I choose the appropriate teaching method to keep the students interested and increase their learning*” P. 8.

### Setting the rules and regulations of the class

Participants cited keeping respect for the class and students. For example, participant number 8 provided a critical perspective about respect for the student and her mutual respect:

*“If you treat them with respect, they will certainly treat you with respect. I have never seen them cause any disruption in the class.”* P. 8.

Participant number 5 shared an experience about respectfully waking up the student:

*“The student was sleeping in the classroom while teaching and learning was in session, I tried to tell her to go and wash her hands and face”.* P. 5.

Creating a disciplinary framework is another approach which participants applied to classroom management.

*“…From the first day of the class, I announce the rules and regulations as a disciplinary framework in the class and even use the students’ participation in developing this framework.” P.9*.

Participants stated the implementation of some disciplinary action for classroom management. Participant number 2 said that having a private meeting with a disruptive student and applying disciplinary action:

*“I believe that this student has not yet learned education and needs more training, and if the inappropriate behavior of the student disrupts the class, I will require the student to leave the class, or after the class, I will call the student and tell him about his behavior”. P.2*.

## Discussion

Classroom management is a critical aspect of teaching that can greatly impact the success of students. This study elaborates on the experiences of faculty members in managing disruptive behavior in the classroom. One of the extracted themes is managing disruptive behaviors/misbehaviors in the classroom had three sub-categories; The participants showed the reaction to the disruptive behavior as purposeful non-verbal gestures in shutting down the disruptive behavior. Other studies confirmed non-verbal communication is a way to manage students’ misbehavior or give effective feedback [26, 38]. One of the tools that may seem ordinary, but can be very important for teaching and class management is non-verbal communication [39].

The findings suggest that avoiding making quick judgments and experiencing the effectiveness of advice with humor were effective approaches to prevent of misbehaviors in the classroom. They used humor to pass indirect commands to stop students’ misbehaviors. Jeder’s study confirms that using humor in classroom management has some benefits such as creating a positive atmosphere for learning and fun, supporting student engagement with course materials, and making students happier [35].

The participants indicated investigating the cause of disruptive behavior and that they held a private meeting with a disruptive student. Sun’s study reported the teachers cited that talking to students after the class sessions was an excellent opportunity to inculcate suitable values, to assist students in making changes and growing up [26]. Talking with students after class and relationship building were student-centered and helped forge good teacher-student relationships conducive to cultivating student trust and positive behavioral changes [26].

The participants also acted as role models and had close and informal communication in prevention of disruptive behavior. So They were careful about their behaviors to be able to act as a successful role model. They had experienced these two components causing to decrease in disruptive behaviors. Other studies confirmed teachers should strive to be positive role models for their students, treating all students equally and avoiding actions that may lead to negative reactions [40].

One of the teachers’ approaches to respond to student misbehavior is trying to guide the disruptive student including; knowing and understanding the conditions of the students, addressing generational differences, having a fair and equal look at every student, ignoring low-level/minor misbehaviors, and communicating with the disruptive student. In every society, counseling and guidance services are essential components in the management of discipline [41]. It is common in schools to refer disruptive students to specially trained staff (e.g., counselors, social workers, and psychologists) for counseling, and this backup system is specifically designed to support teachers and students [26]. Guidance and counseling are services provided to help students optimally develop their potential [42]. These services require the work of competent and professional consultants to ensure that the services provided achieve the goal of meeting the needs of students in a variety of fields, both personal and social, educational and vocational [43].

Participants experienced the prevention of disruptive behavior comprised of the teacher’s role model, close and informal communication, and avoidance of punishment. Other studies reported that teachers served as role models to guide students emulate societal appraised values and behaviors [26, 44]. Chitiyo and May’s study confirmed that the use of punitive strategies such as suspension and detention has been shown to be ineffective in reducing misbehavior [45].

In the present study, the teachers believed they should avoid punishment interventions and they liked to communicate with students closely and informally to lead students’ behaviors unlike Sun’s study, which was conducted with secondary school teachers in Chinese contexts and reported that teachers believed a blend of positive and punitive strategies was needed for reining or changing students’ misbehavior [26]. Plausibly, the social maturity age category of university students might be the reason for teachers to largely avoid meting obvious punitive strategies in the university context unlike the second cycle institutions. In other words, punishments should be primarily future-oriented and used as tools to promote compliance with social norms and reduce norm violations [46]. This means that face-to-face conversations can increase students’ awareness of their behavior and their autonomy in choosing how to act under the guidance of the teacher.

The majority of the participants emphasized the teachers’ role in trying to guide the disruptive students. They cited the knowing and Understanding of the conditions of the students comprising “addressing generational differences”, “ignoring low-level/minor misbehaviors”, and “communicating with the disruptive student”. Teachers must consider the impact of generational differences, between the teachers and students, on the learning environment and classroom management [47]. The generation gap, as another aspect of the learning environment, is a potential cause of increased stress and other experiences of disrespect in nursing education [44].

As mentioned above, the participants tried to guide the disruptive student through a student-centered strategy such as holding a private session with the disruptive student to assess and understand the underlying reasons for manifested misbehavior. Therefore, teachers managed student behavior by building up fair communication. Sun’s study revealed the interviewed teachers confirmed the key to managing misbehavior is good communication with their students [26].

In the present study, the participants provided a protected and safe atmosphere in a classroom environment in which students feel free of any disparities and unhealthy. They paid attention to a fair and equal look at every student, culturally responsive class management, and created a safe and healthy atmosphere. Fallon, et al. reported that teachers’ classes, with higher socio-cultural responsiveness, were with low occurrences of students’ misbehaviors [48]. Darawshe’s study confirmed that every student should be treated as a person, not a jar that needs to be filled with knowledge. Another point of importance was to avoid “student-centered humor” and " discriminatory treatment based on unfair prejudice” [49]. Astor, et al. reported the lack of supportive norms, relationships, and structures in schools/classrooms, makes students more likely to experience violence, peer bullying, and punitive discipline, often accompanied by high levels of absenteeism and poor academic performance [50]. Therefore, creating a safe atmosphere in the classroom environment could be an effective approach to prevent students’ misbehavior.

The participants cited notices and warning compromised of timely feedback/directed commands to manage students’ behavior. Sun’s study reported that teachers usually make specific, directive statements to stop students’ misbehaviors and maintain classroom learning [26]. Directive orders are warnings or threats to force students to follow the teacher’s expectations or rules.

The participants cited encouragement and motivation as another effective strategy to manage students’ misbehavior. They mentioned encouraging students in front of the crowd and praising active students as a key. Allday’s study confirmed a strategy to notice positive behavior in others, teachers need to find good Students who deserve encouragement, are close to misbehaving students, and teachers praise and give benefit to them [38].

The participants cited trying to increase learning and making the class more interesting/attractive. They tried it by the scientific richness of the teacher and choosing the proper teaching method. So that they should have the ability to respond to students’ scientific questions and choose the proper teaching method based on the knowledge of students, class atmosphere, and type of content in choosing a teaching method. Gordon’s study reported that teachers who truly know the content of their lessons and are effective managers of instructional material can then focus on motivational strategies, student assessment, and reflection on teaching and learning [51]. Therefore, novice teachers and experienced teachers if needed, should be trained to manage the classroom and apply all ethical conducts of their profession. They should be prepared and equipped to manage the classroom according to the changes of the present age.

In addition, the participants of the present study set the rules and regulations of the class through keeping respect for the class and students, creating a disciplinary framework/disciplinary action. Sun’s study confirmed that most teachers reported that they first set rules in the classroom, informing students about expected behavior and providing guidelines to follow [26]. Thilagaratnam and Yamat’s study confirmed that teachers agreed on a set of rules that would positively influence students’ behavior, establishing them as the basis for discipline in language classrooms [52].

The main limitation of this study was the possible localization of the findings given that the entire research was conducted within the remit of a specific discipline and geographic area with a clearly defined culture. This may have implications for the generalizability of the findings. In addition, interviews were conducted in an environment outside of the classroom atmosphere, We, therefore, could not critically observe and appreciate participants’ behavior in natural circumstances Finally, the data for the current study were exclusively based on the participants’ experiences which could be infused with social desirability bias. 

## Conclusions

From the teachers’ point of view, it was not only important to effectively manage disruptive behavior, but also prevent disruptive behavior in the classroom. Participants cited strategies that may facilitate their understanding of the causes of misbehavior and implement strategies to tackle students’ misbehaviors by creating a safe and healthy climate to nurture effective learning by students. 

Policymakers should provide teachers with quality classroom management resources by creating suitable infrastructure. Given the increasing changes in teaching and learning styles, the lack of access to extensive resources and training modules for teachers may lead to poor knowledge and skills development needed to managing students’ misbehaviors. Therefore, they force to use unsuitable/punitive responses to classroom disruptions which may have unappropriated outcomes.

Managers should prioritize to the empowerment of teachers, and the teachers themselves should try to improve their classroom management skills. Definitely, the teachers’ training and practice can improve their abilities in classroom management.

### Electronic supplementary material

Below is the link to the electronic supplementary material.

Supplementary Material 1

## Data Availability

The datasets used and/or analyzed during this study are available from the corresponding author.
